# Transcellular communication at the immunological synapse: a vesicular traffic-mediated mutual exchange

**DOI:** 10.12688/f1000research.11944.1

**Published:** 2017-10-24

**Authors:** Francesca Finetti, Chiara Cassioli, Cosima T. Baldari

**Affiliations:** 1 Department of Life Sciences, University of Siena, via A. Moro 2, Siena, 53100, Italy

**Keywords:** immunological synapse, vesicular traffic, transcellular communication, T-cell activation

## Abstract

The cell’s ability to communicate with the extracellular environment, with other cells, and with itself is a crucial feature of eukaryotic organisms. In the immune system, T lymphocytes assemble a specialized structure upon contact with antigen-presenting cells bearing a peptide-major histocompatibility complex ligand, known as the immunological synapse (IS). The IS has been extensively characterized as a signaling platform essential for T-cell activation. Moreover, emerging evidence identifies the IS as a device for vesicular traffic-mediated cell-to-cell communication as well as an active release site of soluble molecules. Here, we will review recent advances in the role of vesicular trafficking in IS assembly and focused secretion of microvesicles at the synaptic area in naïve T cells and discuss the role of the IS in transcellular communication.

## Introduction

T-cell activation crucially depends on the assembly of a complex supramolecular structure, known as the immunological synapse (IS), at the T-cell interface with the antigen-presenting cell (APC) ^1^. The recognition of cognate peptide ligand associated with major histocompatibility complex (pMHC) on APCs by the T-cell antigen receptor (TCR) results in the coordinate polarization of receptors, adhesion molecules, kinases, cytoskeletal elements, and organelles toward the contact area. Intracellular vesicular traffic plays a pivotal role in this polarized transport and is essential for the assembly and maintenance of the IS ^2^. In addition, expanding evidence indicates that microvesicles, generated and released at the synaptic area, are transferred from the T cell to the APC, where they are able to induce the activation of early signaling pathways ^3, 4^. Microvesicles can carry specific microRNAs (miRNAs) that modulate gene expression patterns in the recipient cell ^5^. Moreover, the synaptic cleft has been reported as a site for trogocytosis, a process exploited by T cells to extract pMHC from the APC during the endocytosis of engaged TCRs, which thereby can sustain signaling at endosomes ^6– 12^. These findings support the notion that the IS acts as a device for transcellular communication. Here, we will discuss the role of the IS in cell-to-cell communication in naïve T cells and focus on vesicular trafficking as the main regulator of this process. The polarized secretion of molecules at the IS by effector cells (that is, for example the lytic enzymes by cytotoxic T cells or immunomodulatory molecules by helper T cells) has been well characterized and described in other excellent reviews ^1, 13, 14^.

## Vesicular trafficking at the immunological synapse

The dramatic rearrangement of molecules occurring during IS assembly leads to the formation of two concentric regions within the synaptic cleft ^1, 15^: the central supramolecular activation cluster (cSMAC), where the TCR and the co-stimulatory receptor CD28 accumulate, and the peripheral SMAC, where a ring of integrin bound to newly polymerized actin filaments helps stabilizing the IS. Molecules with bulky ectodomains are segregated at the outer edge of the IS, known as distal SMAC (dSMAC), by a mechanism that excludes molecules above a size threshold from the contact area ^16– 18^.

The orchestration of signaling by surface receptors as well as signaling molecules that accumulate at the IS area crucially depends on the polarization of the microtubule-organizing center (MTOC) ^19^, which brings the endosomal recycling compartment at the T cell:APC contact region, thus favoring directional intracellular trafficking ^20^. In naïve T cells, a number of molecules, including receptors (for example, the TCR) and membrane-associated regulators—for example, the lymphocyte-specific protein tyrosine kinase (Lck) and the linker for activation of T cells (LAT)—are clustered to the synaptic area not only from plasma membrane-associated pools but also from intracellular endosomal pools ^2^. A complex machinery is involved in the selective targeting of specific endosomal proteins to membrane domains. Beyond the common basic recycling pathways, which are involved in the traffic of internalized receptors to the plasma membrane through early endosomes (marked by Rab5) and recycling endosomes (marked by Rab4 and Rab11), more specific members of the Rab GTPase family have been identified as important regulators of endosomal traffic at the IS ^21, 22^. The emerging scenario indicates that T-cell activation relies on the synaptic delivery of receptors and signaling mediators through specific subpopulations of recycling endosomes. This notion is remarkably exemplified by the findings of Soares *et al*., who identified at the IS a mosaic of endosomes characterized by the presence of specific synaptic cargo associated with a unique set of traffic regulators and effectors ^23^. For example, LAT is associated with Rab27a ^+^ and Rab37 ^+^ vesicles, and Lck is associated with Rab11b ^+^ vesicles. It was recently reported that the endosomal localization of Lck depends on Rab11 family interacting protein-3 and alterations in its traffic impair TCR signaling, underscoring the importance of the endosomal pool of Lck for T-cell activation ^24^. The TCR is localized in Rab3d ^+^ and Rab8b ^+^ endosomes ^23^ and its traffic to the IS is regulated by Rab29 ^25^, Rab35 ^26^, and Rab8a ^27^. Surprisingly, the components of the intraflagellar transport (IFT) system, which have a well-known function in ciliogenesis, have been reported as essential for the delivery of the TCR and LAT to the IS and T-cell activation, even though lymphocytes are devoid of a primary cilium ^28– 31^. The complexity of the mechanism underlying the endosomal trafficking to the IS is further increased by a variety of regulators of both the microtubule and the actin cytoskeleton as well as components of the machinery involved in vesicle fusion with the synaptic membrane ^2, 32^. Importantly, several vesicle-soluble N-ethylmaleimide-sensitive factor attachment protein receptors (v-SNAREs) and target-SNAREs (t-SNAREs) are recruited to the IS and are implicated in TCR (VAMP3 ^27, 33^, syntaxin4 ^33^, and SNAP23 ^33^) and LAT (VAMP7 ^34^) targeting to the synaptic membrane.

Collectively, these data indicate that intracellular vesicular trafficking is essential for modulating both the intensity and duration of signaling at the IS through the polarized transport of specific molecules to the synaptic cleft.

## Extracellular traffic at the immunological synapse

Emerging evidence indicates that the IS is not only a site of intense intracellular trafficking but also an area of extracellular vesicle release. Based on these observations, it has been proposed that the cSMAC can be divided into two components: the endo-cSMAC, a membrane domain where TCR signaling occurs, and the exo-cSMAC, an extracellular region between the T cell and the APC, which is characterized by the presence of TCR-enriched extracellular vesicles ^35^. Recently, Choudhuri *et al*. reported that internalized ubiquitinated TCRs can be targeted to microvesicles and budded from the plasma membrane, rather than undergo degradation in lysosomes, through the cooperation of the endosomal sorting complexes required for transport I (ESCRT-I) protein Tsg101 and the vacuolar protein sorting 4 ^3^. Remarkably, these ectosomes carrying TCRs act as a useful device for cell-to-cell communication. Indeed, the TCRs carried by extracellular vesicles are able to engage cognate pMHC on the APC surface and this event has a dual effect: on the one hand, it triggers early intracellular signals in APCs, the intensity of which is proportional to the density of pMHC; on the other hand, the rapid endocytosis of TCR:pMHC complexes results in the signaling of the internalized T-cell ectosomes inside the APC ^3^.

In addition to TCR-enriched ectosomes, T cells release a different type of TCR-containing microvesicle upon antigen receptor triggering ^4^. Blanchard *et al*. showed that these microvesicles contain up to 1% of the total CD3ζ, part of which is phosphorylated on tyrosine residues, as well as Src-related tyrosine kinases (Fyn and Lck), the adaptor protein c-Cbl, the C-X-C motif chemokine receptor (CXCR)-4, and adhesion molecules (CD2 and CD18). The microvesicle content suggests that these can be delivered to cells bearing cognate pMHC, thereby becoming a means of cell-to-cell communication ^4^. Although the membrane compartment from which these microvesicles originate remains to be defined, it has been proposed that they may be exosomes, based on their morphology and the expression of the exosomal markers CD63 and CD18 ^4^.

While the ectosomes containing high levels of TCR are generated at the cell surface, the exosomes appear to derive from the multivesicular bodies (MVBs) ^5^. During IS assembly, MTOC polarization allows MVB translocation just beneath the contact region between the T cell and the APC, which appear essential not only for polarized protein recycling but also for the synaptic release of exosomes ^5, 36^. MVB maturation and exosome secretion depend on the activity of diacylglycerol kinase α, which, in turn, regulates protein kinase D 1/2 ^36, 37^. Moreover, even though traffic regulators such as the ESCRT complex, several members of Rab GTPase family, and SNARE proteins are required both for the biogenesis and for the release of exosomes, many aspects of exosome generation are still to be elucidated ^38, 39^. It has been reported that, in Jurkat T cells, similar to cytotoxic lymphocytes, FasL and APO2L/TRAIL localize at MVBs and are secreted in exosomes upon cell stimulation ^40, 41^. The authors have proposed that the release of death ligands may play an important role in the modulation of immune responses under both physiological and pathological conditions, but further studies are required to clarify this issue ^41^. Mittelbrun *et al*. demonstrated that, upon TCR stimulation, microRNA-containing exosomes, generated through membrane budding and scission from MVBs, polarize and fuse with the synaptic membrane, releasing their content into the cognate APC ^5^. Although a controversial issue is whether the low miRNA copy number (1–10) in exosomes is sufficient to elicit a biological response ^42, 43^, it has been reported that the uptake of exosomes by the APC results in the modulation of the expression of specific genes, such as the Sry-box transcription factor 4 ( *Sox-4*), in the recipient cell ^5^. Finally, although the precise mechanism involved in exosome delivery to acceptor cells remains to be clarified, these pieces of evidence highlight the exchange of genetic material mediated by extracellular vesicles at the IS as a strategy for transcellular communication and immune modulation ^44^.

Remarkably, gap junctions have been described at the IS. Of note, the GJ channel-forming protein connexin 43, a protein involved in gap junction assembly, interacts with the epithelial cell-cell junction protein zona occludens-2 (ZO-2) ^45^, which was recently identified as an IS component ^46^. This suggests that ZO-2 could participate in gap junction formation at the IS. Although this type of cell-to-cell connection has been identified as a means for the transfer of genetic information in different cell systems, a direct synaptic transfer of RNAs through gap junctions in T cells remains to be demonstrated. Nonetheless, the presence at the IS of gap junctions ^47, 48^, as well as of invasive T-cell pseudopodia ^49^ and nanotubes ^50^, strongly suggests that additional mechanisms besides microvesicle secretion are likely to be operational to ensure an exchange of soluble molecules between the T lymphocyte and the APC. This may be required for productive T-cell activation, as already documented for gap junctions ^47, 48^.

The intercellular exchange of membrane patches at the IS through phagocytosis during TCR internalization or upon T-cell dissociation from the APC has also been reported ^51^. The process of extraction of surface molecules, known as trogocytosis, leads to the acquisition by T cells of pMHC as well as adhesion and co-stimulatory molecules expressed on APCs ^7^. The acquisition of membrane patches is promoted by TCR triggering and requires R-Ras2/TC21, a member of R-Ras subfamily GTPase, and the small GTPase RhoG ^6, 8– 10^. T-cell uptake of TCR:pMHC complexes results in prolonged antigen presentation that, in turn, determines increased protein phosphorylation and leads to sustained TCR signaling ^11, 12^. Within T cells, the internalized complexes localize in MVBs ^10^ and can undergo either degradation or recycling to the plasma membrane. Interestingly, recycled pMHCs are exposed on the surface of T cells, allowing these to function as APCs, thus allowing them to potentiate the immune response ^6, 10^. The secretion by APCs of pMHC-containing exosomes able to induce T-cell activation *in vitro* has also been documented ^52– 54^.

Recently, the serine protease inhibitor neuroserpin has been reported to polarize and become secreted at the IS, where it can act as a regulator of the proteolytic balance at the synaptic cleft and affect immune cell function ^55, 56^, highlighting a mechanism to keep under check the contents of the synaptic cleft to which both the T cell and the APC contribute.

## Immune escape mediated by targeting of the cellular vesicular machinery

Among the strategies evolved by pathogens to escape from the host immune response, transcellular communication has been shown to be exploited by the human lymphotropic virus HIV-1 to ensure its spread. Specifically, HIV-1 hijacks the polarized vesicular machinery of its host cell for both the assembly and the focused secretion of newly formed virions at the virological synapse (VS), a highly organized contact zone that forms between infected and uninfected CD4 ^+^ T cells ^57^. The VS and the IS share structural similarities, as well as regulators (for example, EWI-2 and α-actinin) ^58^ and TCR signaling components, despite their divergent kinetics in disassembly and intracellular signaling events (for example, PKCθ) ^59^ that lead to specific outcomes. Interestingly, TCR engagement by pMHC leads to the recruitment and the central accumulation of HIV-1 group-specific antigen (GAG) at the IS, resulting in the budding of GAG-containing microvesicles ^3^. Vesicles secreted by HIV-1–infected cells have been found to carry chemokine receptors, such as C-C motif chemokine receptor-5 and CXCR4, which favor their entry in non-permissive cells ^60, 61^. Also, HIV-1 Nef induces the secretion of extracellular vesicles that contain Nef itself ^62, 63^ in addition to interfering with IS assembly by impairing both the intracellular trafficking of TCR, Lck, and LAT and the organization of the actin cytoskeleton ^64– 69^. Although a previous study showed that Nef-containing vesicles might contribute to the depletion of CD4 ^+^ T cells by inducing the apoptosis of bystander non-infected cells ^63^, Nef was recently described as being required for the release of a disintegrin and metalloprotease 17-loaded exosomes, which make quiescent CD4 ^+^ T cells susceptible to HIV-1 infection ^70^. Hence, HIV-1 co-opts the CD4 ^+^ T-cell secretory apparatus not only to promote a direct cell-to-cell transfer of virions but also to modulate the immune response. Interestingly, extracellular vesicles released by APCs also mediate a counter-strategy to protect recipient T cells from HIV-1 by delivering apolipoprotein B editing complex 3G, a key suppressor of HIV-1 replication ^71, 72^.

## Conclusions

**Figure 1.**
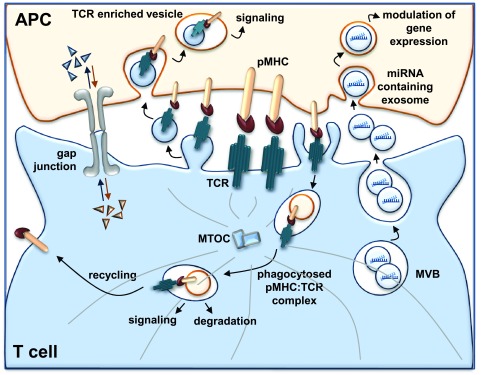
Cell-to-cell communication at the immunological synapse (IS). The IS functions as a device for transcellular communication by exploiting different mechanisms: (i) polarized transfer of T-cell antigen receptor (TCR)-enriched vesicles from T cells to antigen-presenting cells (APCs), which promotes early signaling in the recipient cells; (ii) release of miRNA-loaded exosomes from T cells which modulate gene expression in APCs; (iii) trogocytosis of peptide-major histocompatibility complex:TCR (pMHC:TCR) complexes during TCR internalization, which is associated with both sustained signaling and surface expression of pMHC in T cells, the latter conferring to T cells the ability to present antigen to other T cells; and (iv) gap junction assembly between T cells and APCs that allows the exchange of soluble molecules at the IS. miRNA, microRNA; MTOC, microtubule-organizing center; MVB, multivesicular body.

The IS is a very dynamic structure that must be finely regulated in time and space to induce a productive immune response. Adaptive immunity relies on the correct assembly, maintenance, and disassembly of the IS, which is regulated by TCR and co-stimulatory receptor signaling at the cell surface, intracellular endosomal trafficking, and vesicle secretion at the synaptic cleft. The emerging scenario indicates that the IS is not only a signaling platform but also a device that allows the polarized transfer of molecules or genetic material between T cell and APC ( Figure 1). Even though these mechanisms of transcellular communication may contribute to sustained signaling, thereby potentiating the immune response, the physiological relevance of extracellular vesicle release at the IS remains to be clarified. The improvement of techniques to analyze the IS ^73, 74^ may help elucidate the spatiotemporal dynamics of extracellular vesicles. Moreover, pharmacological treatments as well as genetic manipulations are expected to clarify *in vivo* the role of donor cell–derived microvesicles released into the synaptic cleft in recipient cells as well as the possible connection to disease development.

The IS displays structural and functional similarities to the primary cilium ^2, 75^. Shared regulators of vesicular trafficking are involved in the assembly of these structures. Among these, microtubule-associated protein-4, previously identified as a regulator of cilia formation, has been implicated in MTOC polarization and in the dynamics of signaling nanovesicles during T-cell activation ^76^. Moreover, similar to the IS, the primary cilium has recently been identified as a site for release of active exosomes, and, interestingly, the IFT system is required for the release of extracellular vesicles in *Caenorhabditis elegans* ^77– 79^. This observation, taken together with the requirement for the IFT system in endosomal TCR trafficking and IS assembly, suggests that the IS and the primary cilium may also share regulators of the mechanisms involved in cell-to-cell communication, opening an important area for future research. Remarkably, even though the physiological role of extracellular ciliary vesicles is still unknown, it has been reported that alterations in their release may be linked to ciliary pathologies ^79^. Understanding of the mechanisms involved in the synaptic release and uptake of exosomes can be expected to result in the development of therapeutical applications in the context of immune disorders as well as anti-cancer immunity, as suggested by the tolerogenic effects of vesicles secreted by tumoral cells bearing immunosuppressive molecules ^80, 81^.

## Abbreviations

APC, antigen-presenting cell; cSMAC, central supramolecular activation cluster; CXCR, C-X-C motif chemokine receptor; ESCRT-I, endosomal sorting complexes required for transport I; GAG, group-specific antigen; HIV, human immunodeficiency virus; IFT, intraflagellar transport; IS, immunological synapse; LAT, linker for activation of T cells; Lck, lymphocyte-specific protein tyrosine kinase; miRNA, microRNA; MTOC, microtubule-organizing center; MVB, multivesicular body; Nef, negative regulatory factor; pMHC, peptide-major histocompatibility complex; SNARE, soluble N-ethylmaleimide-sensitive factor attachment protein receptor; TCR, T-cell antigen receptor; VS, virological synapse; ZO-2, zona occludens-2.
